# Two separate endometrioid carcinoma deposits in the colorectum: a rare case report and literature review

**DOI:** 10.3389/fonc.2026.1847045

**Published:** 2026-08-12

**Authors:** Rao Fu, Xinlu Liu

**Affiliations:** First Department of General Surgery, First Affiliated Hospital of Dalian Medical University, Dalian, Liaoning, China

**Keywords:** colorectum, endometrioid carcinoma, endometriosis, malignant transformation, separate lesions

## Abstract

**Background:**

Separate endometrioid adenocarcinoma involving only the gastrointestinal tract is extremely rare. Its diagnosis requires confirmation through pathological examination and immunohistochemical analysis.

**Case:**

We report the case of a nulliparous woman in whom two separate tumour lesions were identified in the rectosigmoid and sigmoid colon, without evidence of malignancy in the uterus or bilateral appendages before surgery. Following laparoscopic radical resection of the colonic lesions and lymph nodes, pathological and immunohistochemical analyses indicated that both lesions represented homogeneous adenocarcinoma of gynaecological origin. Postoperatively, the patient received three months of platinum- and paclitaxel-based chemotherapy. Six months after the initial surgery, the patient underwent a total hysterectomy with bilateral salpingo-oophorectomy, and postoperative histopathological examination again did not reveal any evidence of malignancy in the uterus or bilateral appendages.

**Conclusion:**

This report aims to describe the clinical features, diagnostic challenges, and treatment of this disease based on our case and relevant literature. The optimal treatment strategy for such cases remains to be established.

## Background

Endometrioid carcinoma (EEC) is one of the most common malignancies of the female reproductive tract (1). However, endometrial adenocarcinoma involving the gastrointestinal tract is rare (2), and its occurrence in the absence of any evidence of a gynaecological malignancy is even more uncommon. Consequently, distinguishing such lesions from primary gastrointestinal tumours based solely on morphology can be challenging (3). Pathological examination and immunohistochemical analysis are essential for distinguishing EEC from colorectal adenocarcinoma (CRC) and gastrointestinal stromal tumours (GISTs). Although radical surgical resection remains the preferred treatment approach, the efficacy of subsequent adjuvant therapy requires further study. Here, we report the management of a nulliparous woman who was found to have two EEC lesions in the rectosigmoid and sigmoid colon without evidence of endometrial malignancy in the uterus or bilateral appendages. Postoperative pathological and immunohistochemical findings confirmed the endometrial origin of both lesions.

## Case presentation

A 39-year-old woman (BMI 25.4 kg/m^2^) was admitted to the First Department of General Surgery at the First Affiliated Hospital of Dalian Medical University after a computed tomography (CT) scan performed five months earlier revealed lesions in the bowel. However, she had not experienced abdominal pain, haematochezia, or other symptoms.

The patient underwent hysteroscopic curettage due to irregular vaginal bleeding three months before admission to the hospital, and postoperative pathology suggested atypical endometrial hyperplasia. The patient continued the progestogen treatment for one month. Digital rectal examination revealed no blood or obvious masses in the lower rectum or Douglas cavity. No obvious abnormalities were found on laboratory examination, except for elevated CA125 (60.82 U/mL; normal < 35 U/mL) and HE-4 (102.45 pmol/L; normal < 60.53 pmol/L).

**Figure 1:**
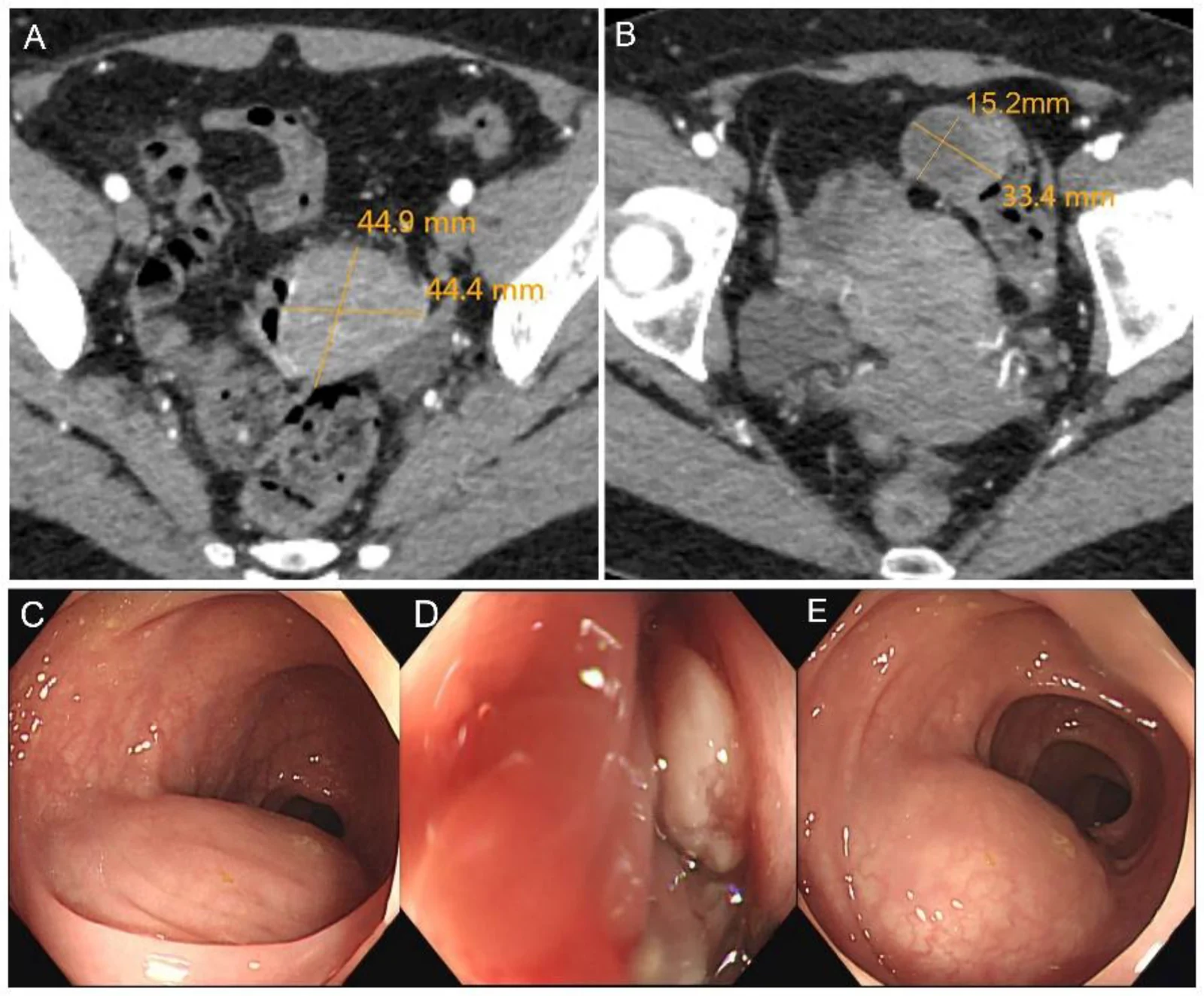
**(A)** Nodular shadow above the uterus with uniform density. The enhanced scan showed a circular, obvious, uneven enhancement. **(B)** Mixed density shadow in front of the uterus with uneven enhancement. **(C, D)** The lesion was located 16 cm from the anus and demonstrated surface ulceration. It compressed the colon cavity and could not be completely visualised. **(E)** A lesion approximately 20 cm from the anus, approximately 3 cm in size, with an unclear boundary and a smooth surface.

Vaginal ultrasound showed a solid mass with rich blood flow signals in the posterior part of the uterus, measuring 4.7 × 5.3 × 4.0 cm, which was suspected to be of gastrointestinal origin. Additionally, a cystic mass was observed near the left adnexal area, measuring 4.4 × 3.6 × 2.9 cm. Contrast-enhanced abdominal CT showed an enhanced uneven nodule shadow above the uterus (**Figure 1A**) and a mixed-density shadow in the front of the uterus (**Figure 1B**). CT angiography (CTA) showed that the lesions were supplied by a branch of the inferior mesenteric artery. It was suspected that the malignant tumour originated from the gastrointestinal tract.

Colonoscopy revealed a submucosal protrusion with ulceration at the rectosigmoid junction (**Figures 1C, D**), and the lumen of the sigmoid colon became narrowed because of compression from the mass (**Figure 1E**). Histopathological examination of the colonoscopic biopsy showed well-organised glands, and that the stroma was scattered with lymphocytes, plasma cells, and a few eosinophils. No clear evidence of malignancy was found.

**Figure 2:**
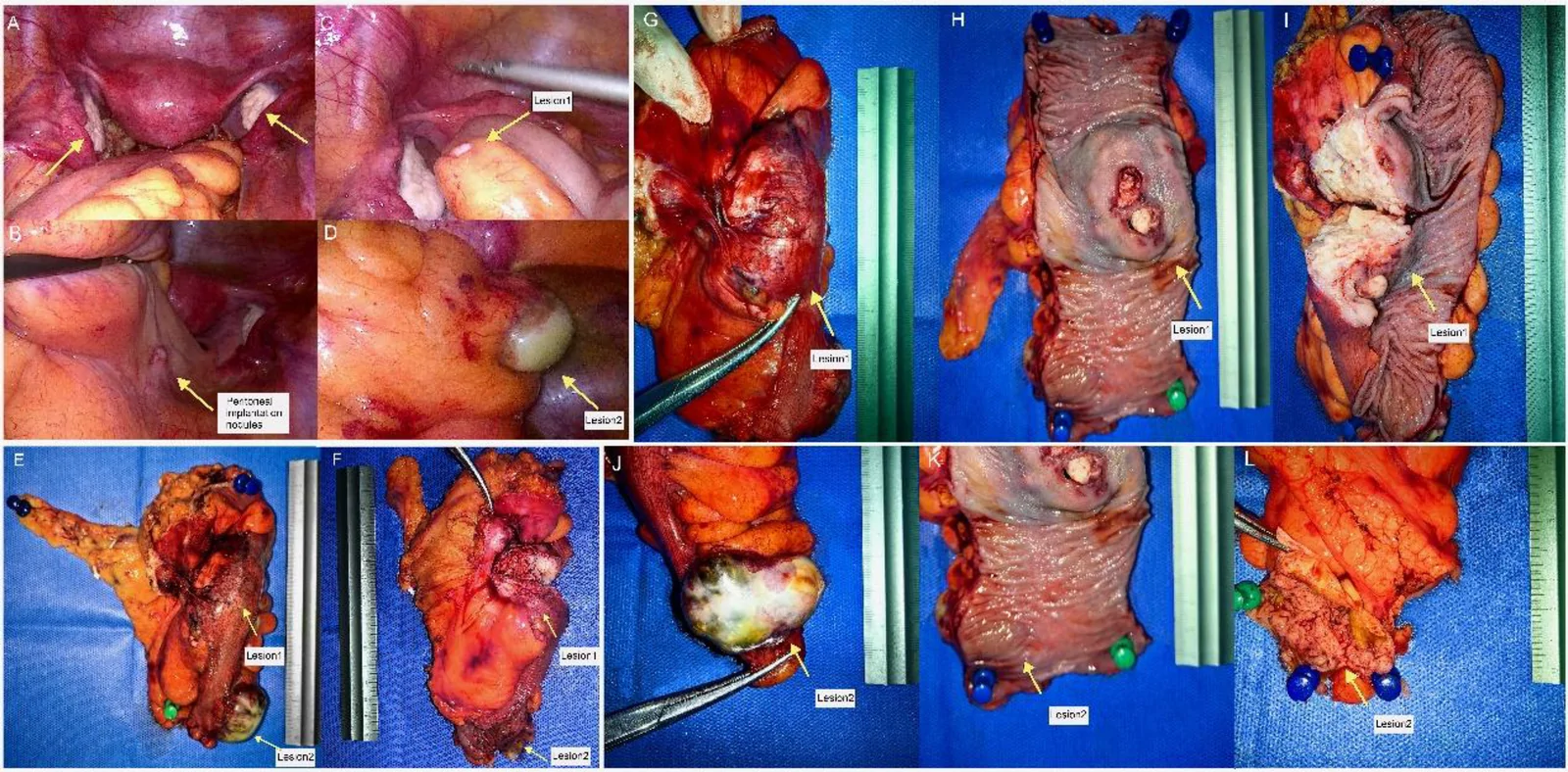
**(A)** Normal uterus and bilateral adnexa, with no chocolate-coloured cysts. **(B)** A grey white hard nodule on the right serosal surface of the sigmoid mesentery, approximately 0.5 cm in size. **(C)** Lesion 1 at the junction of the rectosigmoid; the serosal surface was invaded and appeared greyish-white with an unclear boundary. **(D)** Lesion 2 was a grey-white mass at the sigmoid colon; the surface was smooth with tension and a clear boundary. **(E, F)** Overall appearance of the mucosal and serosal surfaces of two solitary tumours. The distance between these two tumours was 3.5 cm. **(G–I)** Lesion 1 measured 5.2 × 4.5 × 3.5 cm. The cut surface was grey-white, solid, and with a fine texture, involving the whole colon wall, and with an unclear boundary. **(J–L)** Lesion 2 measured 3.5 × 3.5 × 2 cm in the sigmoid colon and involved the whole colon wall.

**Figure 3:**
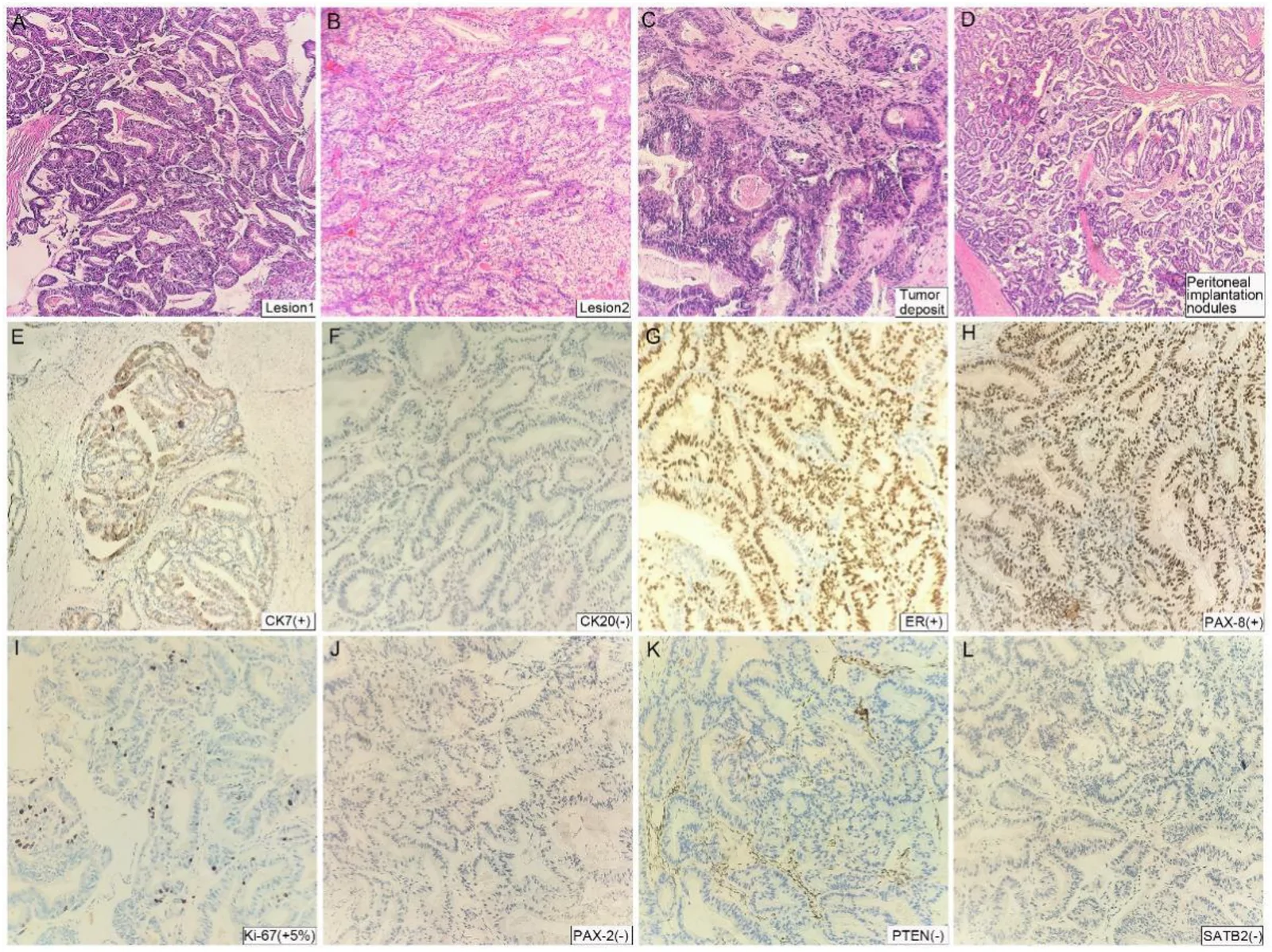
The pathological and immunohistochemical findings (shown at 100x magnification). Examination of the histopathological staining with haematoxylin and eosin (H&E) was performed according to a standard protocol. **(A)** Lesion 1 biopsy showed irregular glandular structures. **(B)** Lesion 2 showed invasive adenocarcinoma with abundant clear cytoplasm. **(C)** Tumour deposits showed adenocarcinoma. **(D)** Peritoneal implantation nodules showed hyperplasia of heterogeneous glands, consistent with adenocarcinoma. **(E)** Positive staining of CK7. **(F)** Negative staining of CK20. **(G)** Positive staining of ER was present in tumour cells. **(H)** Positive staining of PAX-8. **(I)** 5% positive staining of Ki-67. **(J)** Negative staining of PAX-2 in the tumour cells. **(K)** Negative staining of PTEN. **(L)** Negative staining of SATB2.

The patient underwent a laparoscopic exploration one week later. During the operation, the gynaecologist confirmed that the size and appearance of the uterus and bilateral adnexa were normal, and there were no chocolate cysts in the abdominal cavity; therefore, hysterectomy and bilateral salpingo-oophorectomy were not performed (**Figure 2A**). A grey-white, hard nodule (approximately 0.5 cm) was seen on the right serosal surface of the sigmoid mesentery (**Figure 2B**). Intraoperative frozen pathological examination of the nodule suggested adenocarcinoma (**Figure 3D**). Two separate lesions were identified. Lesion 1 was located at the junction of the rectosigmoid, and the serosal surface was invaded and appeared greyish-white with an unclear boundary (**Figure 2C**). Lesion 2 was found in the sigmoid colon, and the grey-white surface was smooth with tension and a clear boundary, although it was difficult to separate from the colon wall (**Figure 2D**). The distance between the two lesions was 3.5 cm (**Figures 2E, F**), with normal colon between them. We performed laparoscopic radical resection of the sigmoid colon and rectosigmoid junction, along with D3 lymph node dissection.

Gross examination of the surgical specimen showed that Lesion 1 measured 5.2 × 4.5 × 3.5 cm, and the cut surface was grey-white, solid, and fine in texture, involving the whole colon wall, with an unclear boundary (**Figures 2G–I**). Lesion 2 measured 3.5 × 3.5 × 2 cm, involving the whole colon wall with a clear boundary. On sectioning, the lesion was cystic and contained dark brown liquid, with white necrotic tissue was visible on the capsule wall (**Figures 2J–L**). Postoperative pathology suggested that both lesions were homogeneous adenocarcinomas, accompanied by vascular invasion. A total of 15 lymph nodes in groups 253, 252, 242, 251, and 241 were negative for metastasis; however, three tumour deposits were identified in groups 251 and 241. (**Figures 3A–C**). Immunohistochemical staining showed CK7(+), CK20(-), ER(+), PAX-8(+), Ki-67(Hot spots+5%), PAX-2(-), PTEN(-), and SATB2(-) (**Figures 3E–L**). The pathology results, combined with immunohistochemistry, indicated EEC.

The patient’s minimal residual disease (MRD) test indicated that the ctDNA was negative after surgery. However, close follow-up remains essential to monitor for recurrence and assess long-term prognosis. The baseline detection of MRD showed that the molecular subtype had no specific molecular profile (NSMP). Genetic analysis identified two Class II somatic PTEN mutations, accompanied by significant copy number loss (39.79% and 31.48%). The patient underwent platinum and paclitaxel chemotherapy, and the follow-up results, including CA125 and HE-4, remained normal. Six months after the initial surgery, the patient underwent a total hysterectomy with bilateral salpingo-oophorectomy in the Gynecology Department. Histopathological examination again revealed no evidence of malignancy, showing only glandular atrophy of the uterus and bilateral appendages, with focal polypoid changes.

## Discussion

EEC confined to the gastrointestinal tract is extremely rare. In this case, despite unremarkable uterine and adnexal findings on preoperative transvaginal ultrasound, hysteroscopic curettage pathology demonstrated atypical endometrial hyperplasia, and the patient presented with early symptoms of irregular vaginal bleeding before surgery, indicating the possibility of occult gynaecological primary endometrial cancer. However, the uniqueness of this case lies in the fact that, even after resection, no evidence of malignant tumours was found in the bilateral adnexal tissues of the uterine fibroids, making this case exceptionally rare.

Postoperative pathology and immunohistochemistry confirmed that the two lesions were independent, homogeneous EEC tissues. Due to the lack of conclusive evidence of malignancy in the uterus and bilateral appendages, only two independent but homogeneous EEC lesions were found. We speculate that the most plausible explanation is the carcinogenesis of ectopic endometrial tissue on the surface of the colon or the implantation of endometrial cancer cells on the surface of the colon, resulting in two separate but homogeneous lesions.

Approximately 0.7%–1% of women with endometriosis (EMT) undergo malignant transformation to EEC, and fewer than 20% of these cases occur at extragonadal sites (4), of which gastrointestinal lesions account for 28% (5, 6). Therefore, malignant transformation of EMT in the gastrointestinal tract to EEC is extremely rare (7). Obesity and oestrogen replacement therapy are clear risk factors for malignant transformation of EMT (8). Studies have indicated that women who have received oestrogen treatment, especially those with a history of EMT, have an increased risk of malignant transformation (9).

The classical Sampson/Scott pathological criteria for the diagnosis of malignant transformation of EMT include the presence of benign endometrial tissues within the tumour, the absence of another primary site of malignancy, and histological transitions between benign and malignant endometriosis. Although no benign endometrial tissue was identified in either lesion in this case, serological findings showed mild-to-moderate increases in serum HE4 and CA125 levels, and genetic analysis revealed two Class II somatic mutations in the PTEN gene. Copy number variation (CNV) analysis revealed a significant reduction in PTEN copy numbers (39.79% and 31.48%), indicative of allelic loss (loss of heterozygosity) and complete functional loss of the PTEN tumour suppressor. These findings suggest the possibility of early driving events in the malignant transformation of endometriosis. The lack of molecular analysis to confirm the clonal origin of the lesions and direct evidence of benign-to-malignant transformation is a limitation of this case.

We reviewed the relevant literature and identified 16 rare cases of EEC arising from gastrointestinal EMT (**Table 1**). The mean age of the patients was 50.43 years. Amongst the sixteen patients, 7 presented with abdominal pain, 7 experienced rectal bleeding, 3 of them also presented with nausea, dysmenorrhea, and intestinal obstruction, and 2 patients presented with pelvic masses. In addition, 8 patients had a history of EMT, 11 underwent hysterectomy, and 6 had also received unopposed oestrogen therapy. The tumours involved the rectum in six cases, the sigmoid colon in five, the small intestine in one, and the rectovaginal septum in one. All patients underwent surgery; six received adjuvant chemotherapy, and two received adjuvant radiotherapy. One patient who received adjuvant radiotherapy died of local pelvic recurrence and liver metastasis. The mean follow-up time without recurrence was 3.4 years.

**Table 1:** Characteristics of patients with endometrioid adenocarcinoma arising from gastrointestinal tract endometriosis.

Number	Age	Signs/symptoms	Location	Tumor markers	Immunohistochemistry	Previous surgery	Hormone therapy	Adjuvant chemotherapy	Adjuvant radiotherapy	Follow-up
1	55	Rectal bleeding and abdominal pain	Rectosigmoid	NA*	ER(+),PAX8(+),CK7(+),CK20(-),CDX2(-)	Excision of bilateral ovarian endometriosis	–	+	–	Rectal recurrence after 22 months
2	75	Rectal bleeding and abdominal pain	Sigmoid colon	NA	CK7(+),CK20(-)	–	–	NA	–	No recurrence 6 months after surgery
3	56	Pain around the anus	Rectum	NA	ER(+),CK7(+),CA125(+),CK20(-),CDX2(-)	Hysterectomy due to uterine myomata	+	–	+	NA
4	49	Pelvic mass	Rectum	CA125elevated	ER(+),PAX8(+),PR(+),CK20(-),SATB2(-),CDX2(-)	–	–	+	–	No recurrence 3 years after surgery
5	38	Hematochezia	Rectum	CA19-9elevated	ER(+),PAX8(+),PR(+),SATB2(+),CDX2(+)	–	–	–	–	No recurrence 1 years after surgery
6	45	Hematochezia and abdominal pain	Rectosigmoid	Normal	ER(+),PR(-),CK7(+),CK20(-)	Ovarian endometrios and operation for rupture of the right ovary.	+	+	–	NA
7	57	Rectal bleeding	Rectum	NA	ER(+),CK7(+),CK20(-)	Hysterectomy with double adnexectomy for pelvic endometriosis and uterine myomatosis	NA	+	–	No recurrence 7 years after surgery
8	52	Rectal bleeding	Sigmoid colon	NA	NA	Hysterectomy and bilateral salpingo-oopherectomy for rectovaginal endometriosis	+	–	–	No recurrence 9 months after surgery
9	27	Abdominal pain and nausea	Sigmoid colon	NA	NA	–	–	NA	–	No recurrence
10	43	Abdominal pain	Sigmoid colon	NA	PAX8(+),CDX2(-)	Hysterectomy with double adnexectomy	–	NA	NA	No recurrence
11	42	Abdominal pain	Rectum	Normal	ER(+),PR(+),PAX8(+),SATB2(-),CDX2(-)	Uterine polypectomy	–	+	–	NA
12	70	Abdominal pain and small bowel obstruction	Sigmoid colon	Normal	ER(+),PAX8(+),CK7(+),CK20(-),CDX2(-)	Uterine fibroids	–	+	–	Rectal recurrence after 22 months
13	55	Melen	Small intestinal		CK7(+),CK20(-)	Hysterectomy and bilateral salpingo-oophorectomy and omentectomy for endometriosi	–	–	–	NA
14	43	NA	Rectovaginal septum	NA	PAX8(+),Ki-67(+)	Right adnexectomy and a left partial ovariectomy for bilateral ovarian endometrioma	+	–	–	No recurrence
15	38	Dysmenorrhea and rectal bleeding	Rectum	NA	ER(+),CD10(+)	Hysterectomy due to uterine fibroid	+	–	–	NA
16	62	Pelvic mass	Rectosigmoid	NA	NA	Hysterectomy and bilateral salpingo-oophorectomy for endometriosis	+	–	+	Local pelvic recurrence and death due to liver metastasis 6 weeks after operation

*not applicable.

The treatment of EEC confined to the gastrointestinal tract has not yet been clearly established. Surgical resection is the standard treatment, but the rarity of cases limits prospective studies evaluating the efficacy of adjuvant therapy, prognosis, and recurrence. Recent trials suggest that external beam radiotherapy (EBRT) and chemotherapy are superior to EBRT or chemotherapy alone (10), and the Statement of the Uterus Commission of the Gynaecological Oncology Working Group (AGO) suggests that combining chemotherapy with immunotherapy results in a significant and clinically relevant improvements in progression-free survival and overall survival (11). Debus et al. suggested that endocrine hormone therapy may also be an option for patients with EEC confined to the gastrointestinal tract (12). However, further clinical studies are needed to confirm these treatment options.

## Data Availability

The original contributions presented in the study are included in the article/supplementary material. Further inquiries can be directed to the corresponding author.
